# Impact of pretreatment with carnitine and tadalafil on contrast-induced nephropathy in CKD patients

**DOI:** 10.1080/0886022X.2019.1669459

**Published:** 2019-12-04

**Authors:** Zaher Armaly, Suheil Artol, Adel R. Jabbour, Amer Saffouri, Nayef Habashi, Amir Abd Elkadir, Naser Ghattas, Raymond Farah, Safa Kinaneh, William Nseir

**Affiliations:** a Department of Nephrology, E.M.M.S. Hospital, and Azrieli Faculty of Medicine in Galilee, Bar- Ilan University, Zafed, Israel;; b Department of Radiology, E.M.M.S. Hospital, Nazareth, Israel;; c Laboratory of Medicine, E.M.M.S. Hospital, Nazareth, Israel;; d Department of Internal Medicine, E.M.M.S. Hospital, Nazareth, Israel;; e Department of Nephrology, HaEmeq Hospital Afula, Afula, Israel;; f Department of Internal Medicine, The Western Galilee Hospital, Nahariya, Israel;; g Department of Internal Medicine “B”, Ziv Medical Center, and Azrieli Faculty of Medicine in Galilee, Bar- Ilan University, Zafed, Israel.

**Keywords:** Radiocontrast, acute kidney injury, carnitine, phosphodiesterase 5 inhibitor, NGAL

## Abstract

**Objective:** The present study assesses whether phosphodiesterase type 5 (PDE-5) inhibitor or carnitine exert nephroprotective effects against clinical contrast-induced nephropathy (CIN).

**Materials and Methods:** The present study consisted of three groups of CKD patients. The first group was control group, who were treated with N-acetyl-L-cysteine 1 day before and on the day of radiocontrast administration. The second one was carnitine group, where the patients were infused with carnitine over 10 min 2 h prior to the radiocontrast administration and 24 h post CT. The third one was PDE-5 inhibitor group, where patients were given tadalafil 2 h prior to the administration of the radiocontrast and in the subsequent day. Urine and blood samples were collected before and at the following time sequence: 2, 6, 12, 24, 48, and 120 h after the contrast administration, for creatinine and NGAL determination.

**Results:** Pretreated with N-acetyl-L-cysteine prior to administration of contrast media (CM) to CKD patients caused a significant increase in urinary but not of plasma neutrophil gelatinase-associated lipocalin (NGAL) and serum creatinine (SCr). In contrast, pretreatment with carnitine prevented the increase in urinary NGAL and reduced SCr below basal levels. Similarly, tadalafil administration diminished the elevation of CM-induced urinary NGAL.

**Conclusions:** These results indicate that carnitine and PDE-5 inhibitors may comprise potential therapeutic maneuvers for CIN.

## Introduction

Imaging procedures such as computer tomography CT or angiography require infusion of iodinated contrast agents intra-arterially or intravenously. While it is a safe procedure among individuals with normal renal function [1,2], it becomes risky in patients with CKD (GFR <60 mL/min) and diabetic patients [3–5]. Contrast-induced nephropathy (CIN) is a common clinical problem, where it becomes the third leading cause of hospital-acquired AKI, accounting for 12–15% of all AKI due to the increasing use of contrast media in diagnostic and interventional procedures, [6–12]. The incidence of CIN is about 20 and 50% in patients with serum creatinine levels above 2 mg/dL and 5 mg/dL, respectively [3–5,10,13]. Additional predisposing risk factors for the development of CIN include diabetic nephropathy, advanced age, congestive heart failure, dehydration, use of NSAIDs or ACE-I, and high or repetitive doses of radiocontrast agent [7,13,14]. The most common clinical course of contrast nephropathy is characterized by a rise in SCr beginning 24–48 h following exposure, peaking within 3–5 days, and resolving within 1–2 week [15]. Besides its high prevalence, the mortality rate in diabetic patient who develops CIN can reach about 30% depending on the patient’s preexisting characteristics and the physicochemical properties of the contrast agent [3,5,7,13,16,17]. Despite the advances in medicine in general and critical care medicine of particular, AKI is still associated with high morbidity and mortality [17–19]. This could be attributed poor understanding of the pathogenesis of CIN, and lack of effective treatment. Specifically, CIN is thought to occur due to a combination of factors, including hypoxia in the renal outer medulla due to perturbations in renal microcirculation and occlusion of small vessels, as well as cytotoxic damage to the tubules directly or via the generation of oxygen free radicals, especially since the concentration of the agent within the tubule is markedly increased. Another factor is transient tubule obstruction with precipitated contrast material [20–26]. Concerning the therapy, several strategies have been suggested to encounter this problem. These include introduction of newer and safer contrast media, improvement of hydration protocols, and the introduction of additional preventive strategies such as N-acetyl-L-cysteine, erythropoietin, antioxidant enzyme mimetics, peroxisome-proliferator-activated receptor agonists, inhibitors of poly(ADP-ribose) polymerase, carbon monoxide-releasing molecules and other measures to enhance hypoxia inducible factors, statins, and adenosine [18,19,27–29]. However, up to date the suggested treatments for CIN are partially effective, emphasizes the need for novel therapeutic strategies for CIN. To the best of our knowledge, the potentially beneficial effects of Carnitine and Phosphodiesterase 5 inhibitor (PDE5-I) on CIN have not yet been examined. However, there is increasing evidence that these agents may be beneficial against CIN in light of their antioxidant and vasodilatory activity [30–34]. Thus, the current study examines whether carnitine or phosphodiesterase type 5 (PDE-5) inhibitor have potential nephroprotective effects against clinical contrast induced nephropathy as compared with N-acetyl-L-cysteine.

## Materials and methods

This was a prospective, non-randomized, open-label, crossover study (randomized, blind) of the effects of I.V. Carnitine supplementation or P.O. tadalafil on renal function and kidney injury in CKD patients who undergo CT imaging that involves administration of radio contrast media. The study was approved by the Nazareth Hospital EMMS Human Research Review Committee and carried at Nazareth Hospital. All patients provided informed consent.

### Inclusion criteria

Patients at age ≥18 years who have been diagnosed as suffering from chronic kidney diseases at stages 3–4 and confirmed by MDRD.

### Exclusion criteria

Pregnant women, CKD patients at stage 5 or on dialysis, Patients with AKI, Patients with severe liver diseases, Patients with severe congestive heart disease, Inter-current illness such as fever, and patients with allergic rhinitis.

### Study design

The demographic and laboratory data of the studied patients are listed in Table 1.

**Table 1.** Demographic characteristics of studied patients.

Parameters/Treatment	N-acetyl-L-cysteine	Carnitine	Tadalafil
Number (*n*)	15	18	12
Age (Year)	73.06 ± 1.63	72.4 ± 1.9	70.8 ± 3.5
Gender Male/Female	60/40%	55.6/44.4%	50/50%
Basal serum creatinine (mg/dL)	1.56 ± 0.12	1.45 ± 0.09	1.73 ± 0.18
Basal MDRD (mL/min)	44.8 ± 3.3	45.8 ± 3.2	37.8 ± 5.9
Haptoglobin Hp1-1/2-1/2-2	7.70/30.80/61.50%	18.78/37.46/43.76%	16.68/41.66/41.66%

The current study included three arms as follows: (1) N-acetyl-L-cysteine group (NAC + S, *N* = 15), in addition to saline, patients were given orally N-acetyl-L-cysteine at a dose of 600 mg twice daily, a day before, on the day of, and 1 day after the contrast administration contrast agent; (2) Carnitine group (Car + S, *N* = 18), in addition to saline, patients will be administered 20 mg/kg carnitine over 20 min 2 h prior to the administration of the contrast agent and 24 h post CT; (3) Phosphodiesterase type 5 inhibitor group (PDE5-I + S, *N* = 12), in addition to saline, patients were given orally 20 mg tablets of tadalafil 2 h prior to the radiocontrast administration and on the subsequent day. Urine and blood samples were collected before and at the following time sequence: 2, 6, 12, 24, 48, and 120 h after the administration of radiocontrast agent. Serum levels of creatinine (SCr) were also determined at 24, 48 and 120 h. CIN was defined as elevation of basal SCr by 0.3 mg/dL or by 50%. Urinary and circulatory levels of NGAL were determined by ELISA, beside Hp genotyping by means of electrophoresis.

### Chemical and haematological analysis

*Determination of NGAL:* This biomarker was determined in specimens of plasma that were stored at −80 °C until analysis. Blood specimens were collected aseptically into EDTA-containing tubes, centrifuged at 3000 rpm and serum separated and stored at −80 °C until testing. Plasma level of NGAL was measured with a commercially available ELISA kit purchased from Bio Porto Diagnostics (Gentofte, Denmark).

*Haptoglobin phenotype:* Haptoglobin phenotype was determined as described by Hochberg et al. [35]. Briefly, serum (10 μL) was mixed with 2 μL of a 10% hemoglobin solution, and the samples were incubated for 5 min at room temperature to permit the haptoglobin-hemoglobin complexes to form. The haptoglobin-hemoglobin complex was resolved by polyacrylamide electrophoresis. The haptoglobin-hemoglobin complexes were visualized by soaking the gel in freshly prepared staining solution. The bands corresponding to the haptoglobin-hemoglobin complex were readily visible within 15 min. All gels were documented with photographs. Phenotypes Hp 1–1, Hp 2–2, and Hp 2–1 were distinguished by a characteristic pattern of bands representing the haptoglobin-hemoglobin complexes.

### Statistical analysis

Data are expressed as means ± SEM. Statistical significance was assessed by one-way analysis of variance (ANOVA) for repeated measures by using Prism 5. Tukey’s multiple comparisons test was used for data point comparisons in each group. *p* ≤ 0.05 was considered statistically significant.

## Results

The average age of the studied three groups of patients was comparable ranging from 73.06 ± 1.63 to 72.4 ± 1.9 years (Table 1). The male/female ratio was 60/40% in the N-acetyl-L-cysteine subgroup, 55.6/44.4% in the carnitine subgroup and 50/50% in the tadalafil subgroup. The basal SCr was 1.56 ± 0.12 mg/dL, 1.45 ± 0.09 mg/dL, and 1.73 ± 0.18 mg/dL in the N-acetyl-L-cysteine, carnitine and tadalafil subgroups, respectively. The basal eGFR according to MDRD formula was 44.8 ± 3.3 mL/min, 45.2 ± 2.9 mL/min and 37.8 ± 5.9 mL/min in the N-acetyl-L-cysteine, carnitine and tadalafil subgroups, respectively.

**Figure 1.**
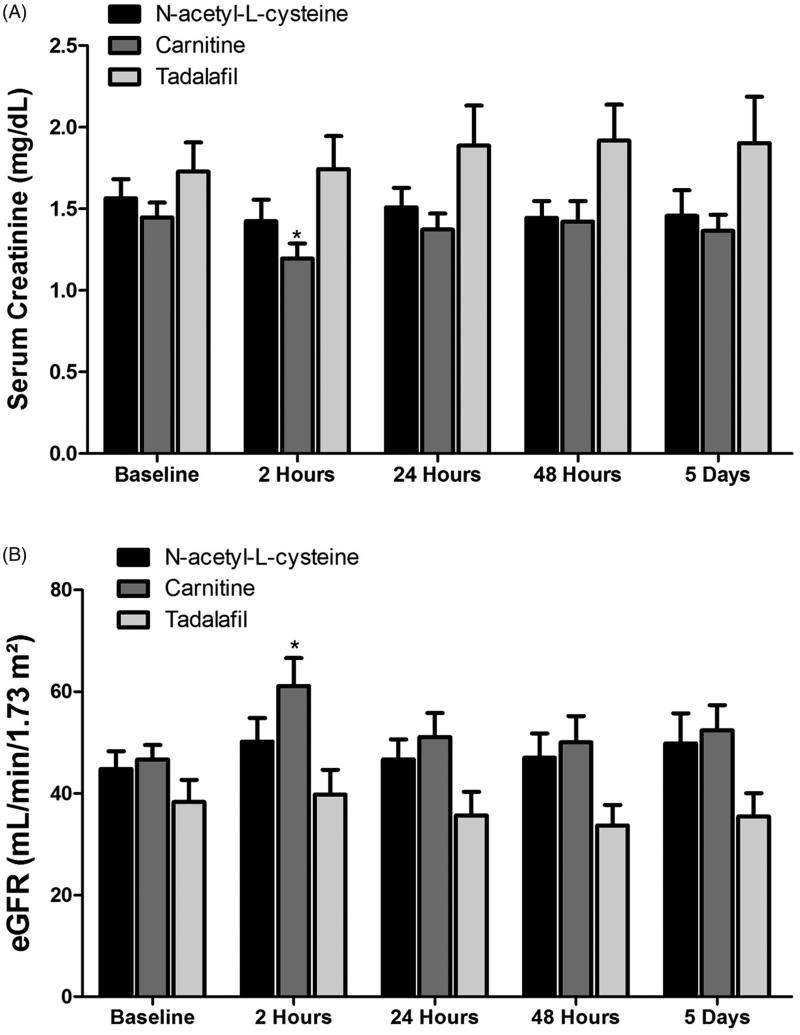
Effects of N-acetyl-L-cysteine, Carnitine and tadalafil therapy on serum creatinine (SCr, A) and estimated GFR (eGFR, B) in patients with CKD (*n* = 12–18) who were administered with radiocontrast as part of CT imaging. Scr levels were determined in blood samples drawn before and 2, 24, 48, 120 h after radiocontrast administration.

The impact of N-acetyl-L-cysteine, carnitine, and tadalafil on SCr and eGFR is depicted in Figure 1. Estimated GFR in patients pretreated with N-acetyl-L-cysteine was not changed throughout the follow up period. When patients were pretreated with carnitine, eGFR significantly increased after 2 h, but returned to basal values after that. Administration of tadalafil to CKD patients that were subjected to CM injection, did not affect neither basal eGFR nor throughout the studied time points.

**Figure 2.**
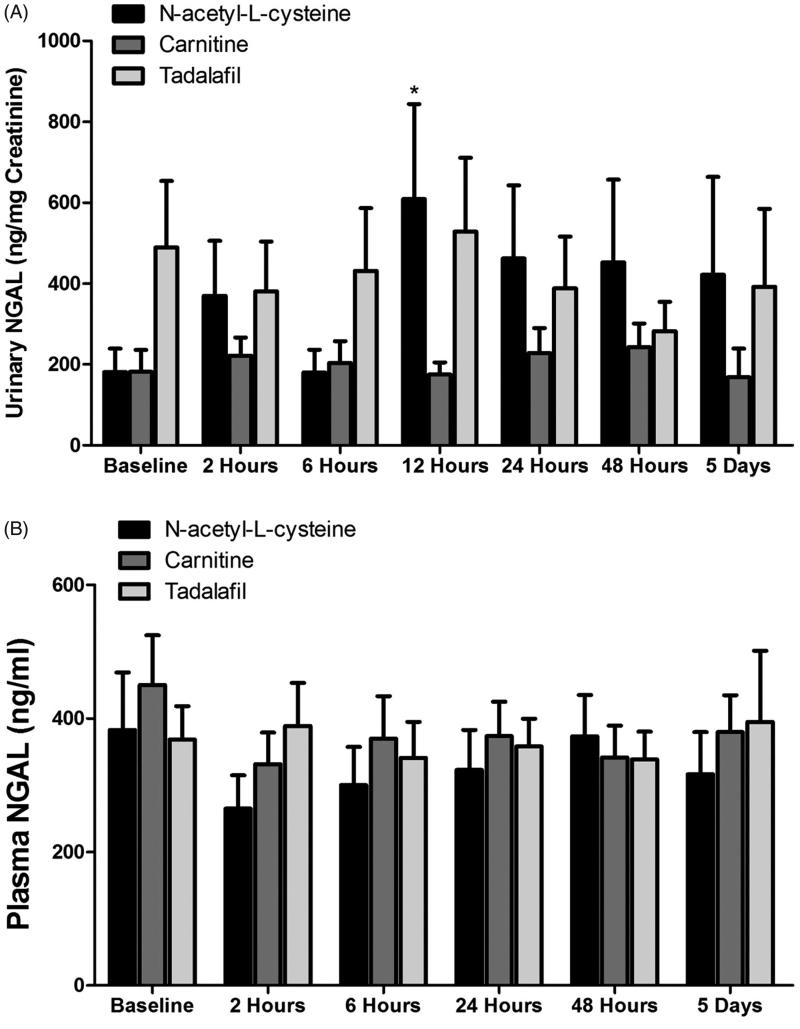
Effects of radiocontrast administration on urinary (A) and plasma (B) NGAL levels in patients with CKD treated with -acetyl-L-cysteine (*n* = 15), Carnitine (*n* = 18) and tadalafil (*n* = 12) prior to intravenous administration of radiocontrast media. Urinary NGAL levels were determined before and 2, 6, 12, 24, 48, 120 h after radiocontrast administration, whereas in the blood before and 2, 24, 48, 120 h after the media injection.

Basal urinary NGAL levels were not statistically different between the studied subgroups, although it was slightly higher in the tadalafil group (*p* = 0.09). Administration of radiocontrast media to patients with CKD who were pretreated with N-acetyl-L-cysteine induced a significant increase in urinary NGAL, but not of plasma NGAL (Figure 2). Specifically, urinary NGAL increased from basal value of 181.1 ± 57.9 ng/mg Creatinine to 608.9 ± 234.9 ng/mg creatinine (*p* < 0.05) after 12 h, and to 462.1 ± 181.1 (*p* < 0.05), after 24 h and remained elevated, although to a lesser extent, after 48 and 120 h (Figure 2). In contrast, pretreatment with carnitine prior to contrast media abolished the increase in urinary NGAL throughout the collection periods in parallel to slight improvement in eGFR and reduction in SCr below basal levels after 2 h (Figure 2). Similarly, administration of tadalafil slightly reduced the elevation in contrast-induced urinary NGAL at 24, 48 and 120 h, but did not affect plasma NGAL (Figure 2). However, it should be emphasized that this subgroup of patients started with higher levels of urinary NGAL as well as SCr and eventually lower eGFR (Figures 1 and 2).

**Figure 3.**
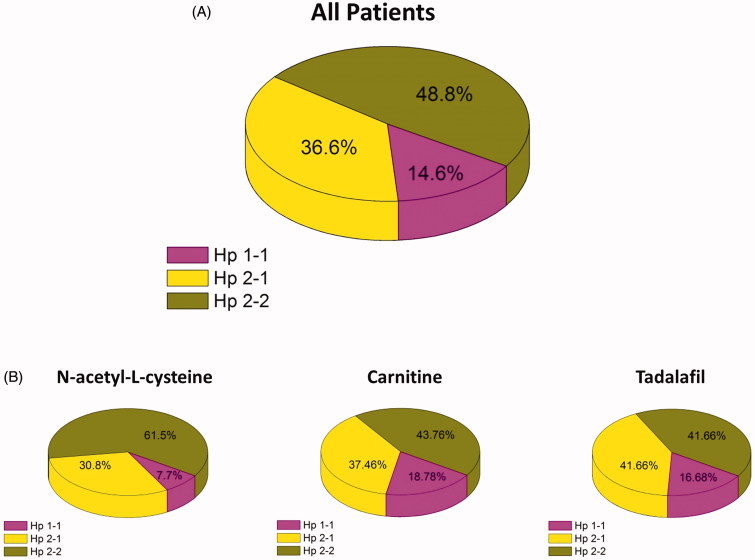
Distribution of haptoglobin phenotype among the studied CKD patients (A) and the various subgroups treated with acetyl-L-cysteine, Carnitine and tadalafil (B).

*Haptoglobin phenotype:* This protocol was designed in order to examine whether Hp phenotype affects contrast-induced kidney dysfunction in CKD patients. Figure 3 depicts the distribution of Hp gene polymorphism among the studied CKD patients in all groups and inside each group. Among all studied patients, Hp1-1, Hp2-1, and Hp 2–2 distribution was 14.6%, 36.6% and 48.8%, respectively. Among the CKD patients in the N-acetyl-L-cysteine group, 7.7% were Hp 1–1, 30.8% were Hp 2–1, and 61.5% were Hp 2–2. In the CKD patients in the carnitine group, 18.78% were Hp 1–1, 37.46% were Hp 2–1, and 43.76% were Hp 2–2. Finally, among the CKD patients in the tadalafil group, 16.68% were Hp 1–1, 41.66% were Hp 2–1, and 41.66% were Hp 2–2.

**Figure 4.**
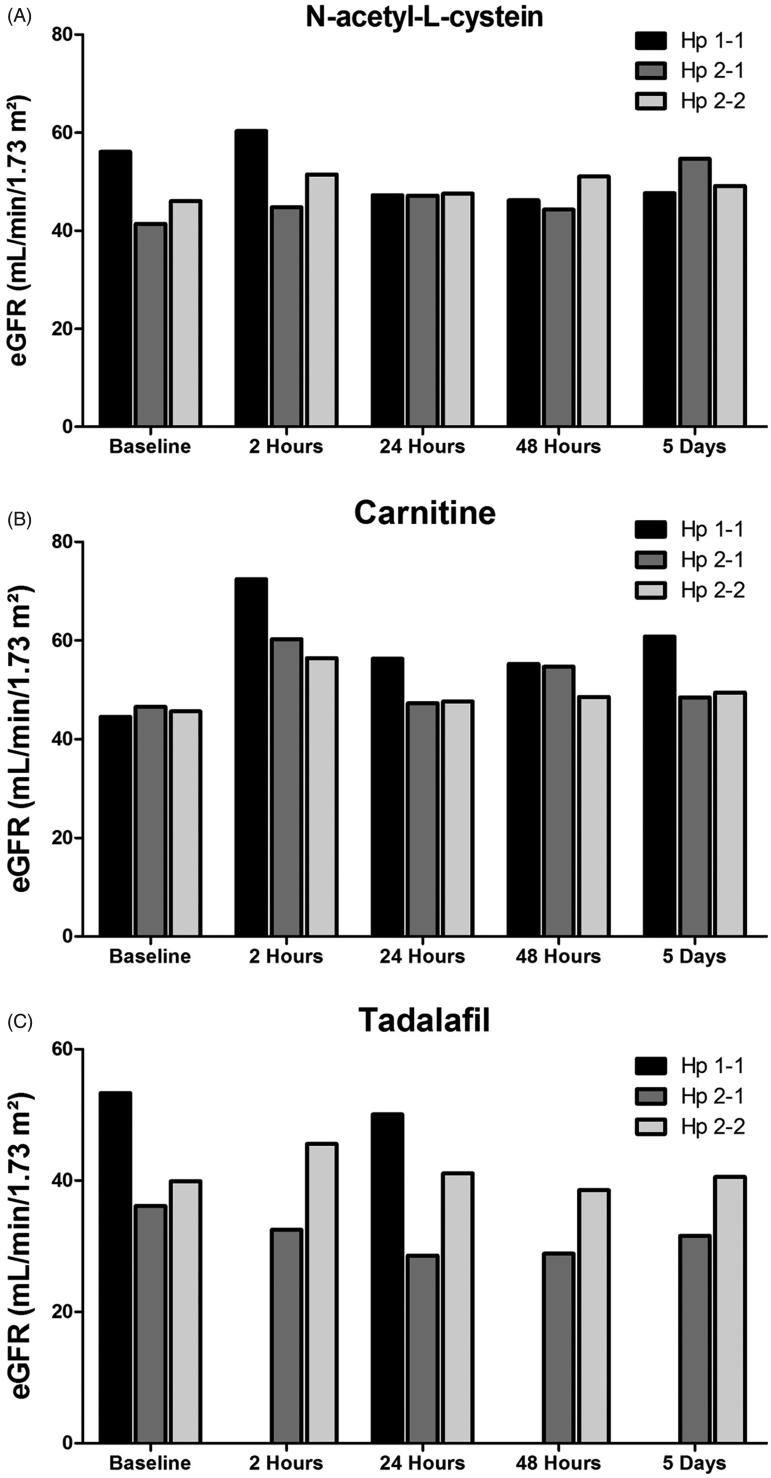
Effect of Haptoglobin genotype on (A) Scr and (B) eGFR in patients with CKD treated with either acetyl-L-cysteine (*n* = 15), Carnitine (*n* = 18) or tadalafil (*n* = 12) prior to intravenous administration of contrast media. Figure 4: Effect of Haptoglobin genotype on (A) urinary NGAL and (B) Plasma NGAL levels in patients with CKD treated with either acetyl-L-cysteine, Carnitine or tadalafil prior to intravenous administration of contrast media. Urinary NGAL levels were determined before and 2, 6, 12, 24, 48, 120 h after radiocontrast administration, whereas in the blood before and 2, 24, 48, 120 h after the media injection.

The impact of Hp genotype on eGFR response to CM in the presence of N-acetyl-L-cysteine, carnitine, and tadalafil pretreatment is presented in Figure 4. Basal eGFR was slightly lower in Hp 2–1 and Hp 2–2 as compared with Hp 1–1. As may be noticed in Figure 4(A), the impact of CM on eGFR in N-acetyl-L-cysteine pretreated patients was deleterious in Hp 1–1 genotype, but not in patients with Hp 2–1 and 2–2, who benefited from such preparation. In Figure 4(B), it is obvious that carnitine pretreatment was nephroprotective in patients with Hp 1–1 and Hp 2–1 genotypes, but not Hp 2–2. When patients were pretreated with tadalafil, neither Hp 1–1 nor Hp 2–1 were benefited from this pretreatment, but Hp 2–2 patients did (Figure 4(C)).

**Figure 5.**
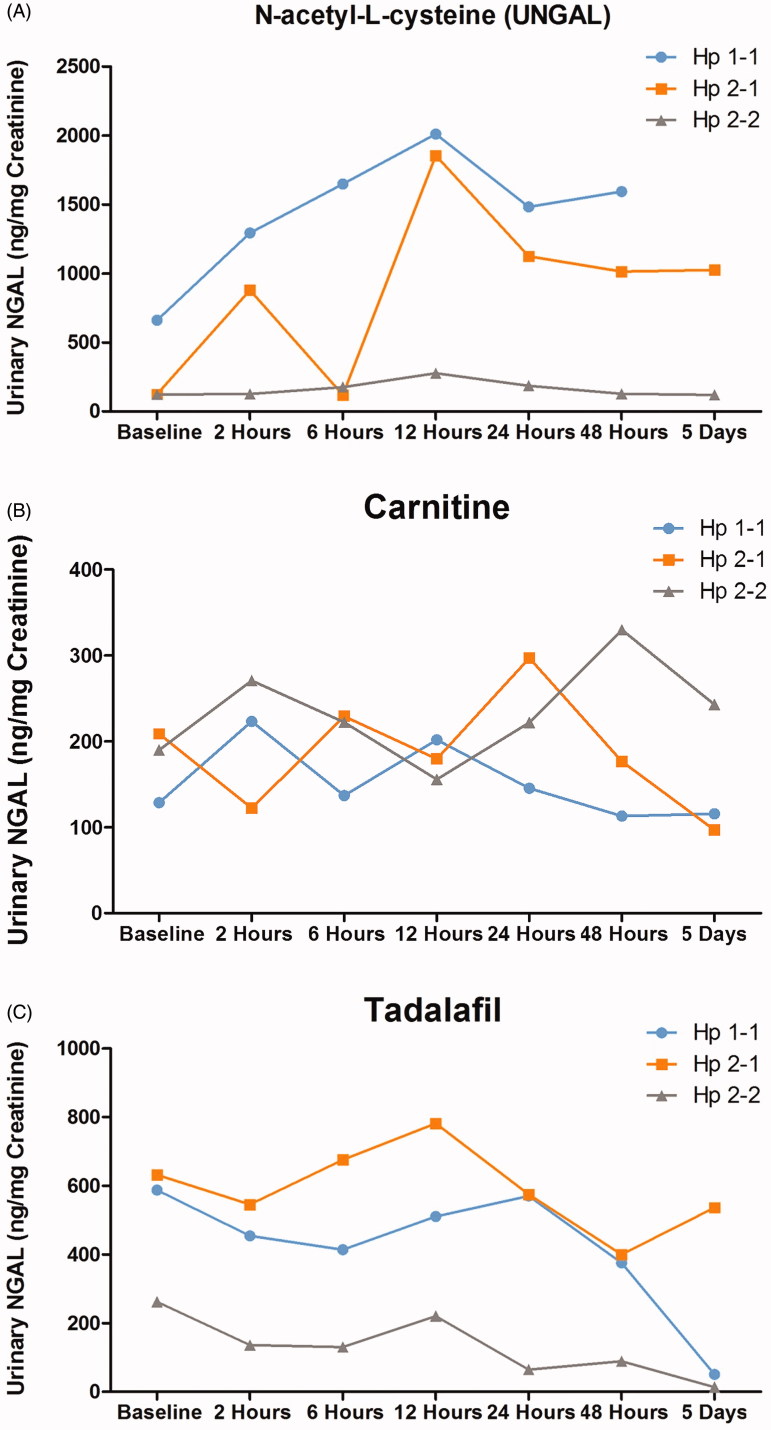
Effect of Haptoglobin genotype on (A) urinary NGAL and (B) Plasma NGAL levels in patients with CKD treated with either acetyl-L-cysteine (*n* = 15), Carnitine (*n* = 18) or tadalafil (*n* = 12) prior to intravenous administration of contrast media. Urinary NGAL levels were determined before and 2, 6, 12, 24, 48, 120 h after radiocontrast administration, whereas in the blood before and 2, 24, 48, 120 h after the media injection.

The alterations in urinary NGAL in response to IV radiocontrast injection in the various subgroups of patients according to their Hp phenotype are depicted in Figure 5. As can be noticed, the increase in urinary NGAL in response to radiocontrast administration in the presence of N-acetyl-L-cysteine pretreatment was more prominent in patients with Hp 1–1 and Hp 2–1 genotype throughout the experiment, but not in Hp-2–2 (Figure 5(A)). Carnitine therapy prevented the increase in urinary NGAL in all groups of Hp genotypes (Figure 5(B)). Interestingly, tadalafil not only prevented the increase in urinary NGAL excretion in Hp 1–1 and Hp 2–1 subgroups of patients administered with IV radiocontrast, it decreased U_NGAL_ regardless the Hp genotype (Figure 5(C)).

## Discussion

So far, there is no preventive prophylactic therapy for CIN. The main approach relies on monitoring renal function as reflected by SCr and eGFR values before and once daily for 5 days after the radiographic procedure in patients at risk especially those with impaired kidney dysfunction and diabetic subjects. In addition, it is recommended to discontinue potentially nephrotoxic medications, such as NSAIDs, diuretics, sodium glucose cotransporter-2 (SGLT2) inhibitors, to apply low volume of radiocontrast media, to encourage oral or intravenous hydration, and to administer N-acetyl-L-cysteine into high-risk patients [15]. The nephroprotective efficacy of additional pharmacological prophylaxis for CIN was tested including antioxidant strategy, inhibition of renal vasoconstriction, as monotherapy or in combination [27]. Likewise, pharmacological interventions with furosemide, mannitol, calcium channel blockers, dopamine, and fenoldopam, did not yield significant benefit for the prevention of CIN and even in certain circumstances aggravated renal damage [36–38]. Regardless the sporadic minor beneficial effects of N-acetyl-L-cysteine, ascorbic acid (vitamin C), and statins, currently there is no well-established pharmacological prophylactic approach to prevent the development of CIN in patients at high risk. As mentioned before, this discouraging could be attributed to our poor understating of the cellular and molecular mechanisms underlying CIN. The latter is largely attributed to: i. reduction in medullary blood flow which leads to hypoxia, ii. Direct tubule cell damage and iii. The formation of reactive oxygen species [15,27].

The present study provides new insights into the mechanisms underlying renal damage in response to intravenous administration of radiocontrast into CKD patients and the potential nephroprotective effects of either carnitine or PDE-5 inhibitors against CIN in these patients. We showed that administration of radiocontrast to patients with CKD, who were pretreated with N-acetyl-L-cysteine caused a significant increase in urinary NGAL, but not of plasma NGAL and SCr, probably secondary to extensive hydration that may dilute the plasma and subsequently masking potential elevated circulatory NGAL or SCr. In contrast, when these patients were pretreated with carnitine prior to CM, urinary NGAL was not increased during the follow-up time. In addition, carnitine therapy reduced SCr below basal levels after few hours from the contrast injection. Similarly, administration of tadalafil attenuated contrast-induced elevation in urinary NGAL, but did not affect neither plasma NGAL nor Scr. These findings indicate that carnitine and PDE-5 inhibitors may comprise potential prophylactic therapies for contrast-induced nephropathy.

The nephroprotective, at least at the injury level, of carnitine and tadalafil are not surprising in light of the reported anti-oxidative and anti-inflammatory properties of these compounds. In this context, experimental studies shows that L-propionylcarnitine, a propionyl ester of L-carnitine, was able to prevent cyclosporine-induced acute nephrotoxicity, reducing lipid peroxidation, lowering blood pressure and preventing renal hypofiltration in animals treated with cyclosporine for long-term [39]. Patients treated with carnitine displayed improved physical performance and treatment-related chronic fatigue, cardiovascular disease, cancer, diabetes, and other chronic syndromes, caused by impaired carnitine production in kidney disease [30]. In the last decade there are increasing reports describing the beneficial use of carnitine for a better energy metabolism (mitochondrial metabolism), increases albumin and protein levels, restores antioxidant defenses, improving nutritional status, cardiac, vascular smooth muscle, and muscular function [40]. The postulated beneficial effect of carnitine treatment is by directing lipids towards oxidation and ATP production. Another possible protective effect of carnitine on contrast media induced lesions is its ability to suppress the development of oxidative stress and free radical generation [31]. Free radicals, and in particular hydroxyl radical, lead to lipid peroxidation of cell membranes, causing degradation of phospholipids results in increased production of vasoconstrictors [41], what might be another indirect factor stands behind contrast media induced vasoconstriction. In patients with CKD, the carnitine balance and mitochondrial β-oxidation are disruptive, mainly due to the decline of the GFR [42]. This may cause several metabolic disturbances at the cellular level, including impaired mitochondrial fatty acid oxidation and energy production, accumulation of toxic acyl moieties, and inhibition of key enzymes of metabolic pathway [43]. These metabolic abnormalities may lead to the several clinical alterations often observed in these patients, such as muscle weakness and myopathy, loss of body protein and cachexia, insulin resistance and glucose intolerance, plasma lipid abnormalities, anemia refractory to erythropoietin treatment, cardiomyopathy, and intradialytic symptoms [44,45], thus may explain the higher risk these patients are found for contrast-induced AKI, as was evident by enhanced urinary NGAL excretion.

Concerning phosphodiesterase 5 inhibitor, there is increasing evidence that this family, including sildenafil, vardenafil and tadalafil, have broader effects than vasodilation, most likely due their ability to inhibit the breakdown of cGMP, the second messenger of nitric oxide and natriuretic peptides [33,46]. Since, experimental studies have demonstrated that PDE5 inhibitors, improve endothelial function [47], and reduce remarkably the infarct size in rat model of myocardial infarction [48,49], it is appealing to assume that these inhibitors may possess nephroprotective effects in AKI setting too, including contrast-induced renal injury. Indeed, few studies have demonstrated that PDE5 inhibitors exert renal beneficial effects in I/R rat model [50,51], and post cardiopulmonary bypass AKI in swine [52]. While, these studies examined the effect of pretreatment PDE-5 inhibitors such as tadalafil and sildenafil on renal histology, oxidative stress or kidney function, none of them examined the impact of carnitine or PDE-5 inhibitors on the more sensitive biomarkers of AKI, namely NGAL and KIM-1, in AKI setting. Therefore, the present study expand previous ones by examining whether tadalafil or carnitine exerts nephroprotective effect in CKD patients undergoing CT imaging which involves CM administration, by measuring kidney function, urinary NGAL, besides SCr and eGFR. Our results clearly demonstrate that both tadalafil and carnitine abolished the elevation in urinary NGAL excretion in patients administered with CM, suggesting a nephroprotective effect of these agents against contrast media-induced AKI. The mechanisms underlying the beneficial effects of PDE5 inhibitors in CKD patients could be attributed to improvement in endothelial function, attenuation of the inflammatory status and oxidative stress, a hallmark feature in CKD.

Unfortunately, the interaction between the Hp genotype and CIN was not studied in predialytic CKD patients. Thus, the current study is the first, to the best of our knowledge, to examine whether Hp genotyping affects the renal susceptibility o CM on one hand and which subgroup of patients may enjoy potential nephroprotective effects of tadalafil or carnitine. It has been shown that the Hp 1–1 protein is superior to the Hp 2–2 protein in this antioxidant function [53,54]. In line with this assumption, longitudinal cohort studies have shown that diabetic patients carrying the Hp2-2 type are more vulnerable for oxidative stress injury [55–57]. In agreement with this notion and the inferiority of Hp 2–2 as antioxidant, we demonstrated that patients who were pretreated with N-acetyl-L-cysteine, carnitine, or tadalafil displayed attenuated urinary NGAL excretion response to CM, as compared with other Hp genotypes.

Despite the findings of the current study it has few limitations such as low number of patients in each group, using a single marker of AKI, i.e., NGAL, using patients who received NAC as control group rather than saline, and there was no differentiation between CKD subgroups due to the low number of patients.

In summary, as expected administration of radiocontrast into patients with CKD induced kidney injury as was evident by enhanced urinary excretion of NGAL/When these patients were pretreated with carnitine or PDE-5 inhibitors this CM-induced deleterious effect was abolished. Moreover, patients who are of Hp 2–2 genotype may benefit from this maneuver as compared with Hp 1–1 and Hp2-1, suggesting carnitine and tadalafil (PDE-5 inhibitor) may serve a prophylactic therapy approaches for CIN, especially in patients with Hp 2–2 genotype.

## References

[1] Solomon R Contrast-Induced Acute Kidney Injury (CIAKI). Radiol Clin North Am. 2009;47(5):783–788. 19744593 10.1016/j.rcl.2009.06.001

[2] Curtis LM, Agarwal A HOpe for contrast-induced acute kidney injury. Kidney Int. 2007;72(8):907–909. 17914415 10.1038/sj.ki.5002530

[3] Tumlin J, Stacul F, Adam A, et al. Pathophysiology of contrast-induced nephropathy. Am J Cardiol. 2006;98(6):14K–20K. 16949376 10.1016/j.amjcard.2006.01.020

[4] Oudemans-van Straaten HM Contrast nephropathy, pathophysiology and prevention. Int J Artif Organs. 2004;27(12):1054–1065. 15645616 10.1177/039139880402701208

[5] Tepel M, van der Giet M, Schwarzfeld C, et al. Prevention of radiographic-contrast-agent–induced reductions in renal function by acetylcysteine. N Engl J Med. 2000;343(3):180–184. 10900277 10.1056/NEJM200007203430304

[6] Hou SH, Bushinsky DA, Wish JB, et al. Hospital-acquired renal insufficiency: a prospective study. Am J Med. 1983;74(2):243–248. 6824004 10.1016/0002-9343(83)90618-6

[7] McCullough PA, Wolyn R, Rocher LL, et al. Acute renal failure after coronary intervention: incidence, risk factors, and relationship to mortality. Am J Med. 1997;103(5):368–375. 9375704 10.1016/s0002-9343(97)00150-2

[8] Gruberg L, Mehran R, Dangas G, et al. Acute renal failure requiring dialysis after percutaneous coronary interventions. Catheter Cardiovasc Interv. 2001;52(4):409–416. 11285590 10.1002/ccd.1093

[9] Rudnick M, Feldman H Contrast-induced nephropathy: what are the true clinical consequences? CJASN. 2008;3(1):263–272. 18178787 10.2215/CJN.03690907

[10] Rihal CS, Textor SC, Grill DE, et al. Incidence and prognostic importance of acute renal failure after percutaneous coronary intervention. Circulation. 2002;105(19):2259–2264. 12010907 10.1161/01.cir.0000016043.87291.33

[11] Levy EM, Viscoli CM, Horwitz RI The effect of acute renal failure on mortality. A cohort analysis. JAMA. 1996;275(19):1489–1494. 8622223

[12] Marenzi G, Lauri G, Assanelli E, et al. Contrast-induced nephropathy in patients undergoing primary angioplasty for acute myocardial infarction. J Am Coll Cardiol. 2004;44(9):1780–1785. 15519007 10.1016/j.jacc.2004.07.043

[13] McCullough PA, Adam A, Becker CR, et al. Risk prediction of contrast-induced nephropathy. Am J Cardiol. 2006;98(6A):27K–36K. 10.1016/j.amjcard.2006.01.022 16949378

[14] Kagan A, Sheikh-Hamad D Contrast-induced kidney injury: focus on modifiable risk factors and prophylactic strategies. Clin Cardiol. 2010;33(2):62–66. 20186983 10.1002/clc.20687 PMC6653592

[15] Andreucci M, Solomon R, Tasanarong A Side effects of radiographic contrast media: pathogenesis, risk factors, and prevention. Biomed Res Int. 2014;2014:741018. 24895606 10.1155/2014/741018 PMC4034507

[16] Reddan D, Fishman EK Radiologists’ knowledge and perceptions of the impact of contrast-induced nephropathy and its risk factors when performing computed tomography examinations: a survey of European radiologists. Eur J Radiol. 2008;66(2):235–245. 17728089 10.1016/j.ejrad.2007.05.012

[17] Chertow GM, Burdick E, Honour M, et al. Acute kidney injury, mortality, length of stay, and costs in hospitalized patients. JASN. 2005;16(11):3365–3370. 16177006 10.1681/ASN.2004090740

[18] Schrier RW, Wang W, Poole B, et al. Acute renal failure: definitions, diagnosis, pathogenesis, and therapy. J Clin Invest. 2004;114(1):5–14. 15232604 10.1172/JCI22353 PMC437979

[19] Lameire N, Van Biesen W, Vanholder R Acute renal failure. Lancet. 2005;365(9457):417–430. 15680458 10.1016/S0140-6736(05)17831-3

[20] Nash K, Hafeez A, Hou S Hospital-acquired renal insufficiency. Am J Kidney Dis. 2002;39(5):930–936. 11979336 10.1053/ajkd.2002.32766

[21] Katzberg RW, Haller C Contrast-induced nephrotoxicity: clinical landscape. Kidney Int. 2006;69:S3–S7. 10.1038/sj.ki.5000366 16612398

[22] Briguori C, Marenzi G Contrast-induced nephropathy: pharmacological prophylaxis. Kidney Int. 2006;69:S30–S38. 10.1038/sj.ki.5000372 16612399

[23] Goldenberg I, Matetzky S Nephropathy induced by contrast media: Pathogenesis, risk factors and preventive strategies. CMAJ. 2005;172(11):1461–1471. 15911862 10.1503/cmaj.1040847 PMC557983

[24] Mehran R, Nikolsky E Contrast-induced nephropathy: definition, epidemiology, and patients at risk. Kidney Int. 2006;69:S11–S15. 10.1038/sj.ki.5000368 16612394

[25] Tepel M, Aspelin P, Lameire N Contrast-induced nephropathy: a clinical and evidence-based approach. Circulation. 2006;113(14):1799–1806. 16606801 10.1161/CIRCULATIONAHA.105.595090

[26] Finn WF The clinical and renal consequences of contrast-induced nephropathy. Nephrol Dial Transplant. 2006;21(6):i2–i10. 16723349 10.1093/ndt/gfl213

[27] Hung Y-M, Lin S-L, Hung S-Y, et al. Preventing radiocontrast-induced nephropathy in chronic kidney disease patients undergoing coronary angiography. WJC. 2012;4(5):157–172, 22655164 10.4330/wjc.v4.i5.157 PMC3364502

[28] Spandou E, Tsouchnikas I, Karkavelas G, et al. Erythropoietin attenuates renal injury in experimental acute renal failure ischaemic/reperfusion model. Nephrol Dial Transplant. 2006;21(2):330–336. 16221709 10.1093/ndt/gfi177

[29] Agarwal A, Nick HS Renal response to tissue injury: lessons from heme oxygenase-1 GeneAblation and expression. J Am Soc Nephrol. 2000;11(5):965–973. 10770977 10.1681/ASN.V115965

[30] Fabio Di Lisa NS, Barbato R, Menabò R Carnitine and mitochondrial dysfunction in carnitine today. Springer US; 1997.

[31] Dokmeci D, Akpolat M, Aydogdu N, et al. The protective effect of L-carnitine on ionizing radiation-induced free oxygen radicals. Scand J Lab Anim Sci. 2006;33(2):75–83.

[32] Armaly Z, Abd El Qader A, Jabbour A, et al. Effects of carnitine on oxidative stress response to intravenous iron administration to patients with CKD: impact of haptoglobin phenotype. BMC Nephrol. 2015;16(1):135. 26268514 10.1186/s12882-015-0119-0 PMC4535251

[33] Reffelmann T, Kloner RA Phosphodiesterase 5 inhibitors: are they cardioprotective? Cardiovasc Res. 2009;83(2):204–212. 19474180 10.1093/cvr/cvp170

[34] Armaly Z, Abassi Z Phosphodiesterase-5 inhibitors : potential nephroprotective agents. Clin Exp Pharmacol. 2014;4(3):1–5.

[35] Hochberg I, Roguin A, Nikolsky E, et al. Haptoglobin phenotype and coronary artery collaterals in diabetic patients. Atherosclerosis. 2002;161(2):441–446. 11888529 10.1016/s0021-9150(01)00657-8

[36] Cacoub P, Deray G, Baumelou A No evidence for protective effects of nifedipine against radiocontrast-induced acute renal failure. Clin Nephrol. 1988;29:215–216. 3365867

[37] Solomon R, Werner C, Mann D, et al. Effects of saline, mannitol, and furosemide on acute decreases in renal function induced by radiocontrast agents. N Engl J Med. 1994;331(21):1416–1420. 7969280 10.1056/NEJM199411243312104

[38] Stone GW, McCullough PA, Tumlin JA, et al. Fenoldopam mesylate for the prevention of contrast-induced nephropathy: a randomized controlled trial. JAMA. 2003;290(17):2284–2291. 14600187 10.1001/jama.290.17.2284

[39] Bertelli A, Giovannini L, Palla R, et al. Protective effect of L-propionylcarnitine on cyclosporine-induced nephrotoxicity. Drugs Exp Clin Res. 1995;21(6):221–228. 8907697

[40] Evangeliou A, Vlassopoulos D Carnitine metabolism and deficit–when supplementation is necessary? CPB. 2003;4(3):211–219. 10.2174/1389201033489829 12769764

[41] Parra T, De Arriba G, Arribas I, et al. Cyclosporine a nephrotoxicity: role of thromboxane and reactive oxygen species. J Lab Clin Med. 1998;131(1):63–70. 9452128 10.1016/s0022-2143(98)90078-6

[42] Fabio Di Lisa NS, Barbato R, Menabò R Carnitine and carnitine esters in mitochondrial metabolism and function In: De Jong JW, Ferrari R, editors. The carnitine system; a new therapeutical approach to cardiovascular diseases. Dordrecht (The Netherlands); Boston (MA): Kluwer Academic Publishers; 1995 p. 21–38.

[43] Borum PR Clinical aspects of human carnitine deficiency. New York (NY): Pergamon; 1986.

[44] Massry SG, Kopple JD Nutritional management of renal disease -carnitine in renal failure. Lippincott Williams & Wilkins; 1997.

[45] Guarnieri G, Toigo G, Crapesi L, et al. Carnitine metabolism in chronic renal failure. Kidney Int Suppl. 1987;32:S116–S127. 3323608

[46] Sandner P, Hütter J, Tinel H, et al. PDE5 inhibitors beyond erectile dysfunction. Int J Impot Res. 2007;19(6):533–543. 17625575 10.1038/sj.ijir.3901577

[47] Rosano GMC, Aversa A, Vitale C, et al. Chronic treatment with Tadalafil improves endothelial function in men with increased cardiovascular risk. Eur Urol. 2005;47(2):214–222. 15661417 10.1016/j.eururo.2004.10.002

[48] Ockaili R, Salloum F, Hawkins J, et al. Sildenafil (Viagra) induces powerful cardioprotective effect via opening of mitochondrial K _ATP_ channels in rabbits. Am J Physiol Heart Circ Physiol. 2002;283(3):H1263–H1269. 12181158 10.1152/ajpheart.00324.2002

[49] Sesti C, Florio V, Johnson EG, et al. The phosphodiesterase-5 inhibitor tadalafil reduces myocardial infarct size. Int J Impot Res. 2007;19(1):55–61. 16858368 10.1038/sj.ijir.3901497

[50] Sohotnik R, Nativ O, Abbasi A, et al. Phosphodiesterase-5 inhibition attenuates early renal ischemia-reperfusion-induced acute kidney injury: assessment by quantitative measurement of urinary NGAL and KIM-1. Am J Physiol Ren Physiol. 2013;304(8):F1099–F1104. 10.1152/ajprenal.00649.2012 23364806

[51] Guzeloglu M, Yalcinkaya F, Atmaca S, et al. The beneficial effects of tadalafil on renal ischemia-reperfusion injury in rats. Urol Int. 2011;86(2):197–203. 21160160 10.1159/000321927

[52] Patel NN, Lin H, Toth T, et al. Phosphodiesterase-5 inhibition prevents postcardiopulmonary bypass acute kidney injury in swine. Ann Thorac Surg. 2011;92(6):2168–2176. 21983073 10.1016/j.athoracsur.2011.07.002

[53] Melamed-Frank M, Lache O, Enav BI, et al. Structure-function analysis of the antioxidant properties of haptoglobin. Blood. 2001;98(13):3693–3698. 11739174 10.1182/blood.v98.13.3693

[54] Bamm VV, Tsemakhovich VA, Shaklai M, et al. Haptoglobin phenotypes differ in their ability to inhibit heme transfer from hemoglobin to LDL. Biochemistry. 2004;43(13):3899–3906. 15049697 10.1021/bi0362626

[55] Milman U, Blum S, Shapira C, et al. Vitamin E supplementation reduces cardiovascular events in a subgroup of middle-aged individuals with both type 2 diabetes mellitus and the haptoglobin 2-2 genotype: a prospective double-blinded clinical trial. ATVB. 2008;28(2):341–347. 10.1161/ATVBAHA.107.153965 18032779

[56] Levy AP, Gerstein HC, Miller-Lotan R, et al. The effect of vitamin E supplementation on cardiovascular risk in diabetic individuals with different haptoglobin phenotypes. Diabetes Care. 2004;27(11):2767. 10.2337/diacare.27.11.2767 15505023

[57] Blum S, Vardi M, Brown JB, et al. Vitamin E reduces cardiovascular disease in individuals with diabetes mellitus and the haptoglobin 2-2 genotype. Pharmacogenomics. 2010;11(5):675–684. 20415560 10.2217/pgs.10.17 PMC2880717

